# 基于毛细管电泳和离子迁移率经验公式测定洛伐他汀的绝对淌度和解离常数

**DOI:** 10.3724/SP.J.1123.2021.01014

**Published:** 2021-12-08

**Authors:** Fang LUO, Zehua GUO, Chengxi CAO, Liuyin FAN, Wei ZHANG

**Affiliations:** 1.上海交通大学生命科学技术学院, 微生物代谢国家重点实验室, 上海 200240; 1. School of Life Science and Biotechnology, State Key Laboratory of Microbial Metabolism, Shanghai Jiao Tong University, Shanghai 200240, China; 2.上海交通大学电子信息与电气工程学院, 上海 200240; 2. School of Electronic Information & Electrical Engineering, Shanghai Jiao Tong University, Shanghai 200240, China; 3.上海交通大学学生创新中心, 上海 200240; 3. Student Innovation Center, Shanghai Jiao Tong University, Shanghai 200240, China

**Keywords:** 毛细管区带电泳, 经验方程, 淌度, 解离常数, 洛伐他汀, capillary zone electrophoresis (CZE), empirical equation, mobility, dissociation constant, lovastatin

## Abstract

作为一种可以预防动脉粥样硬化和冠心病的潜在药物洛伐他汀,其绝对淌度*m*_0_和解离常数p*K*_a_值的测定有助于其性质与应用的研究。在前期相关研究基础上,该文提出了一种基于毛细管区带电泳(CZE)和离子淌度经验公式测定洛伐他汀*m*_0_和p*K*_a_的新方法。首先,根据经验公式由实际淌度(*m*_act_)、有效淌度(*m*_eff_)和*m*_0_之间的关系推导出*m*_0_的计算公式。对于一元酸HA,根据之前*m*_0_的计算公式,以氢离子的浓度为自变量,*m*_eff_的倒数为因变量可得到一条直线。根据这条直线的斜率计算得到p*K*_a_。为了验证该方法的可行性和可靠性,应用该方法测定了巴比妥酸、苯甲酸、苄胺、苯酚、间甲酚等有机酸碱的*m*_0_和p*K*_a_值。同时,对于pH值低于6的缓冲体系,采用反向毛细管电泳技术,测定其p*K*_a_,并将测得的实验结果与理论参考值进行对比,发现两者具有较高的一致性,*m*_0_的标准偏差小于6.0%, p*K*_a_的标准偏差小于6.2%,且由线性回归方程的相关系数(*R*)可以看出测定p*K*_a_时的线性回归直线拟合度较好,说明该文建立的新方法具有较高的可靠性。最后基于这种CZE与经验公式结合的新方法,采用二甲基亚砜(DMSO)作为电渗流标记物测定了洛伐他汀的*m*_0_和p*K*_a_,得到的值分别为-1.70×10^-8^ m^2^/(V·s)和9.00。该方法适用于酸性和碱性分析物*m*_0_和p*K*_a_等理化参数的测定,在药物分析尤其是新药理化特性研究中具有重要意义。

## Abstract (en)

In capillary electrophoresis, determination of the basic physical and chemical properties of compounds, such as absolute mobility (*m*_0_) and dissociation constant (p*K*_a_), is of great practical significance. This is because the aforementioned properties are often used for the qualitative or quantitative analyses of the relevant compounds toward their application as potential drugs. Lovastatin is a potential drug candidate that can reduce the levels of cholesterol and low-density lipoprotein cholesterol in the blood, as well as prevent atherosclerosis and coronary heart disease. For a more convenient and rapid investigation of the properties and applications of lovastatin, it is necessary to determine its *m*_0_ and p*K*_a_ values. However, existing research on capillary electrophoresis for lovastatin and other related drugs focus on their quantitative determination, and their action mechanism and functions. Unfortunately, there are very few studies aimed at the determination of the *m*_0_ and p*K*_a_ values of lovastatin. Based on related studies, this paper herein proposed a novel method to determine *m*_0_ and p*K*_a_ of lovastatin. The present study mainly included a calculation method and experimental verification. The calculation method was based on capillary zone electrophoresis (CZE) and the empirical formula of ion mobility. First, on the basis of the empirical formula, the calculation formula for *m*_0_ was derived from the relationship between the actual mobility (*m*_act_), effective mobility (*m*_eff_) and *m*_0_. Second, for a monovalent acid (HA), according to the calculation formula for *m*_0_ part, considering the hydrogen ion concentration as the independent variable and the reciprocal of *m*_eff_ as the dependent variable, a straight line was obtained on the coordinate axis. From the slope of this straight line, the dissociation equilibrium constant *K*_a_ was obtained directly, and p*K*_a_ was calculated easily. After the derivation of *m*_0_ and p*K*_a_ in the theoretical part, the feasibility and reliability of this method were verified by using it to determine the *m*_0_ and p*K*_a_ values of several organic acids and bases (barbituric acid, benzoic acid, benzylamine, phenol, and *m*-cresol) in the experimental part. Note that for the buffer system with pH<6.0, reverse capillary electrophoresis was used for the determination of p*K*_a_, because this technique helped shorten the migration time and facilitates the detection of analytes that could not reach the cathode. After obtaining *m*_0_ and p*K*_a_, the theoretical reference values for these parameters were obtained by PeakMaster 5.1. The experimental data were well consistent with the theoretical *m*_0_ and p*K*_a_ values. The standard deviation (SDs) of *m*_0_ and p*K*_a_ were less than 6.0% and 6.2%, respectively. From the correlation coefficient (*R*) of the linear regression equation, it was found that the linear regression lines of p*K*_a_ fit well, indicating the excellent reliability of this method. Finally, with this simple and reliable method, dimethyl sulfoxide (DMSO) was used as a marker for electroosmotic flow to determine the *m*_0_ and p*K*_a_ values of lovastatin (-1.70×10^-8^ m^2^/(V·s) and 9.00, respectively). This method is suitable for the determination of *m*_0_ and p*K*_a_ of acidic and basic analytes. The method has high accuracy and is expected to play an indispensable role in drug analysis.

近年来,生物化学工程的迅速发展使得大量化合物成为潜在候选药物,了解这些化合物理化性质的需求也日益增长。这也对如绝对淌度(*m*_0_, m^2^/(V·s))和解离常数(p*K*_a_)等理化参数的测定方法提出了更高要求。其中,*m*_0_指化合物在离子强度趋于零的条件下的淌度,而p*K*_a_是水溶液中具有一定解离度的溶质的极性参数。

洛伐他汀是胆固醇合成通路中重要酶羟甲基戊二酰辅酶A还原酶(HMG-CoA还原酶)的抑制剂,可减少血胆固醇和低密度脂蛋白胆固醇的含量,预防动脉粥样硬化和冠心病等^[1]^。洛伐他汀的最大吸收波长*λ*_max_为238 nm,可溶于碱性溶液。洛伐他汀包括内酯型和酸型(见图1),而内酯型洛伐他汀的亲脂性较强,口服吸收率低,须在肝脏中水解成酸型后才能发挥药理作用。

**图1:**
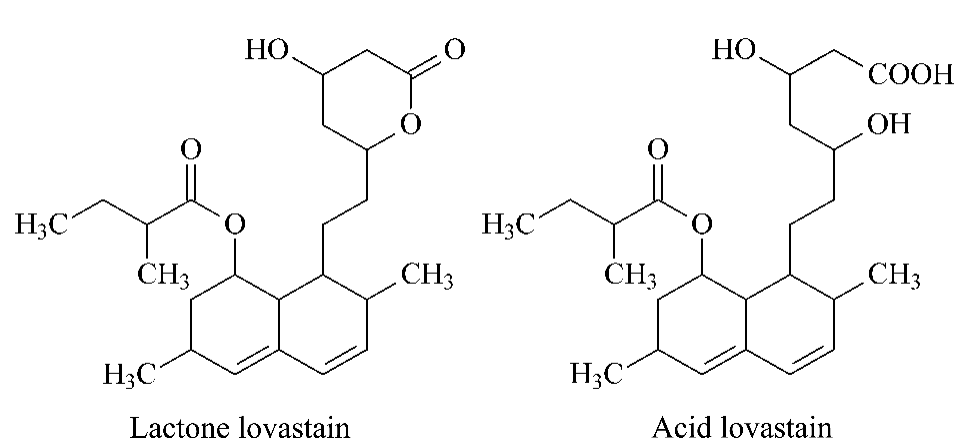
内酯洛伐他汀和酸性洛伐他汀的结构

当环境pH值高于7.7时,内酯型洛伐他汀转化为酸型洛伐他汀^[2]^。因而有必要对洛伐他汀的重要理化参数进行测定,了解其理化特性,从而为更深入的研究提供基本数据和信息。但是,目前大多数对洛伐他汀药物的研究工作主要聚焦于含量测定、作用机理和功能方面,关于测定洛伐他汀*m*_0_和p*K*_a_值的研究则较少^[1,2,3]^。

当前已经有许多关于其他化合物p*K*_a_值的测定方法,其中最经典的方法是电位计滴定法和光谱法^[4,5,6,7,8]^。但这些方法相对来说需要耗费大量样品,并且对于纯度低的化合物测定结果不够准确^[9,10]^。自20世纪70年代以来,高效液相色谱法(HPLC)也已应用于化合物p*K*_a_的测定中^[11,12,13,14,15]^。Bartolini等^[16]^使用反相高效液相色谱法(RP-HPLC)测定乙酰胺的p*K*_a_,但液相色谱方法流动相溶液消耗较多,而且单个样品分析时间至少10 min以上,比较耗时。包括毛细管等速电泳法(ITP)和毛细管区带电泳法(CZE)等在内的毛细管电泳法(CE)也广泛应用于水溶性化合物p*K*_a_值的测定中^[17,18,19,20,21,22,23,24,25]^。

本研究基于CZE和离子淌度的经验方程^[26,27,28]^,建立了一种精确测定洛伐他汀*m*_0_和p*K*_a_的新方法。根据经验方程,可以由测得的实际淌度(*m*_act_, m^2^/(V·s))计算*m*_0_,再通过*m*_0_进一步计算得到p*K*_a_。在方法验证部分,使用该方法测定了巴比妥酸、苯甲酸、苄胺、苯酚、间甲酚等有机酸碱的*m*_0_和p*K*_a_。对pH值低于6的缓冲体系,加入阳离子表面活性剂,采用反向毛细管电泳技术,测定其p*K*_a_值。将测定值和理论参考值进行比较,结果发现二者一致性较高,说明该方法具有较高的可靠性。最后应用该方法测定洛伐他汀的*m*_0_和p*K*_a_。

## 1 理论推导

### 1.1 *m*_0_的计算方法

在CZE中,有效淌度的计算方法如下^[29]^:


(1) ${{m}_{eff}}={{m}_{app}}-{{m}_{EOF}}=\frac{{{L}_{\text{tot}}}{{L}_{\text{eff}}}}{V}\left( \frac{1}{{{t}_{\text{obs}}}}-\frac{1}{{{t}_{\text{EOF}}}} \right)$

其中,*m*_eff_和*m*_app_分别表示分析物的有效淌度和表观淌度(m^2^/(V·s)), *m*_EOF_表示其电渗流淌度(m^2^/(V·s)), *L*_tot_和*L*_eff_分别为毛细管的总长度和有效长度(m), *V*表示所施加的电压(V), *t*_obs_是观察到的分析物迁移时间(s), *t*_EOF_是电渗流下观察到的中性标记物迁移时间(s)。

对于一价弱酸来说,*m*_act_与*m*_eff_之间的关系为^[30]^:


(2) ${{m}_{eff}}={{m}_{act}}\alpha$

其中,*m*_act_是实际淌度,即在给定的离子强度下完全质子化或去质子化离子的淌度(m^2^/(V·s))。*α*是解离度。对于一价酸HA,离解度可定义为:


(3) $\alpha =\frac{{{C}_{\text{HA}}}-\left[ \text{HA} \right]}{{{C}_{\text{HA}}}}\text{=}\frac{\left[ {{\text{A}}^{-}} \right]}{{{C}_{\text{HA}}}}$

式中,*C*_HA_是反应开始时一元酸的浓度(mol/L), [HA]、[A^-^]和[H^+^]分别是反应达到平衡时溶液中一元酸HA、A^-^和H^+^的浓度。当分析物100%电离时,其解离度*α*趋于1,此时式(2)可写为:


(4) ${{m}_{eff}}={{m}_{act}}$

一价离子*m*_0_的经验公式^[26-28,30-32]^可以写成:


(5) ${{m}_{act}}={{m}_{0}}exp(-\eta \sqrt{zI})$

其中,*η*为常数,当电荷数*z*为1时(即一元酸或碱), *η*为0.5030;当*z*大于等于2时,*η*为0.6629。*I*为离子强度,根据以下公式来计算:


(6) $I=1/2\sum {{c}_{i}}z_{i}^{2}$

式中,*c_i_*和*z_i_*代表组分*i*的浓度(mol/L)和电荷数。将式(4)代入式(5)可得*m*_0_为:


(7) ${{m}_{0}}=\frac{{{m}_{\text{act}}}}{\text{exp}\left( -\eta \sqrt{zI} \right)}\text{=}\frac{{{m}_{\text{eff}}}}{\text{exp}\left( -\eta \sqrt{zI} \right)}$

需要注意,等式(5)和(7)仅适用于*I*≤0.1 mol/L和25 ℃条件下。

综上,当分析物处于完全解离状态时,全部以离子形式存在,溶液中无分析物的分子形态。因此可以根据分析物的p*K*_a_理论参考值,选择合适pH值的缓冲液以确保分析物能完全解离。在固定的pH下,可以得到分析物在特定离子强度下的*m*_act_,并根据公式(7)计算分析物在不同离子强度下的*m*_0_。

### 1.2 p*K*_a_的计算方法

对于一元酸HA来说,解离平衡常数为:


(8) ${{K}_{a}}=\frac{\left[ {{\text{H}}^{+}}\left] \cdot \right[{{\text{A}}^{-}} \right]}{\left[ \text{HA} \right]}$

将式(3)代入式(8),得:


(9) ${{K}_{a}}=\frac{\alpha \left[ {{\text{H}}^{+}} \right]}{1-\alpha }$

由式(9)进一步可得:


(10) $pH=p{{K}_{a}}+log\frac{\alpha }{1-\alpha }$

根据式(10),如果溶液的pH值比酸性(或碱性)分析物的p*K*_a_值大(或小)3,即log (*α*/(1-*α*))的值为3时,经计算*α*为0.999,则分析物处于完全解离状态,此时满足1.1节中*m*_0_的测定条件。结合公式(2)和(9),可得:


(11) $\frac{1}{{{m}_{\text{eff}}}}\text{=}\frac{1}{{{m}_{\text{act}}}}+\frac{\left[ {{\text{H}}^{+}} \right]}{{{m}_{\text{act}}}{{K}_{\text{a}}}}$

由式(11)可知,1/*m*_eff_与[H^+^]成正比,可通过计算pH值得到。当离子强度一定时,在不同的pH值下可以得到一系列*m*_eff_值。以氢离子浓度为自变量(*x*), 1/*m*_eff_为因变量(*y*),得到一条线性回归直线。该直线的斜率1/(*m*_act_*K*_a_)。由斜率和公式(5)中计算得到的*m*_act_,可以求得分析物的*K*_a_,从而进一步求得其p*K*_a_。

## 2 实验部分

### 2.1 仪器与试剂

高效毛细管电泳仪(1229型,北京新技术仪器有限公司);未涂覆熔融石英毛细管(总长50 cm(有效长度为25 cm和40 cm)、内径75 μm,中国河北永年光纤厂);超纯水系统(德国SG Water公司)用以生产电导率低至0.055 μS/cm的超纯水;pH计(瑞士Mettler Toledo公司)。

氢氧化钠、磷酸氢二钠、磷酸三钠、磷酸二氢钠、二甲基亚砜(DMSO)、乙酸、醋酸钠、丙酮、巴比妥酸、苯甲酸、苯酚、间甲酚等试剂均购自上海化学试剂公司;苄胺(纯度>99%)购自美国Fluka公司;十六烷基三甲基溴化铵(CTAB)(纯度>99%)购自中美生物科技有限公司;洛伐他汀片(20 mg/片)购自浙江海正股份有限公司。

### 2.2 背景电解质溶液的制备

对于*m*_0_的测定,制备表1所示的一系列pH值相同而离子强度不同的磷酸盐缓冲液,每种缓冲液均制备5种离子强度(0.01、0.04、0.05、0.09和0.1 mol/L)。

**表1:** 测定*m*_0_所用缓冲液

Buffer system	pH	Analyte
Na_2_HPO_4_-NaH_2_PO_4_	8.00	barbituric acid
Na_2_HPO_4_-NaH_2_PO_4_	8.00	benzoic acid
Na_2_HPO_4_-NaH_2_PO_4_	6.00	benzylamine
Na_2_HPO_4_-NaOH	12.50	phenol
Na_2_HPO_4_-NaOH	12.50	m-cresol

All pH were measured at 25 ℃.

对于p*K*_a_的测定,制备表2所示的一系列离子强度相同(0.05 mol/L)而pH值不同的磷酸盐和醋酸盐缓冲液。所有缓冲液用0.45 μm过滤器过滤并保证在两周内使用。

**表2:** 测定p*K*_a_所用缓冲液

Buffer system	pH		I/(mol/L)
Na_2_HPO_4_-NaOH	12.50-	12.00	0.05
Na_2_HPO_4_-Na_3_PO_4_	11.00-	9.00	0.05
Na_2_HPO_4_-NaH_2_PO_4_	8.00-	6.00	0.05
NaAc-HAc	5.00-	4.00	0.05

*I*: ionic strength. All pH were measured at 25 ℃

### 2.3 样品的制备

巴比妥酸、苯甲酸、苄胺、苯酚、间甲酚样品分别以1、30、10、0.8、2 mg/mL溶于纯水中。用相应缓冲液将样品分别稀释至终浓度为0.02、0.06、2、0.08、0.10 mg/mL后进样。洛伐他汀样品以1 mg/mL溶解于75%乙醇中,用对应缓冲液稀释至200 μg/mL后进样。

### 2.4 毛细管预处理及电泳条件

每次进样前将毛细管依次用1.0 mol/L NaOH、超纯水和1.0 mol/L HCl分别冲洗20、10和20 min,最后用该样品对应的缓冲液冲洗30 min。当缓冲液的pH值等于或低于6.0时(本实验中为乙酸缓冲液),在缓冲体系中加入0.8%(v/v)阳离子表面活性剂CTAB,并用含有CTAB的缓冲液冲洗毛细管10 min,然后进行电泳实验。

实验控制在25 ℃室温下进行,设置分离电压为10~15 kV,并以1.33 kPa的压力从毛细管阳极端(在常规CZE下)或从毛细管阴极端(反向CZE下)注入样品。进样过程持续10 s,根据分析物的性质选择合适的吸收波长(巴比妥酸和间甲酚用214 nm,苯甲酸、苄胺和苯酚用254 nm)。使用二甲基亚砜(214 nm紫外吸收波长)或丙酮(254 nm紫外吸收波长)作为中性标记物来标记电渗流(EOF)。

## 3 结果与讨论

### 3.1 不同分析物*m*_0_和p*K*_a_的测定结果

实验结果根据1.1节和1.2节推导的公式,计算得到不同分析物的*m*_0_和p*K*_a_实验测定值,同时使用通用软件PeakMaster 5.1计算得到其对应*m*_0_和p*K*_a_的理论参考值(见表3)。测定值与理论参考值相减得到差值,该差值与理论参考值的比值为标准偏差(SD)。从计算结果可以看出,对于*m*_0_和p*K*_a_的测定,其SD分别不超过6.0%和6.2%。在p*K*_a_的测定中,线性回归方程相关系数(*R*)的范围为0.940~0.999,表明该线性回归直线拟合度较好。这说明该方法得到的*m*_0_和p*K*_a_测定值与理论参考值一致度较高,证明该测量方法是可靠的,可以用于分析物*m*_0_和p*K*_a_的测定。

**表3:** *m*_0_和p*K*_a_的实测值和参考值

Analyte	m_0_×10^-8^/(m^2^/(V·s))			pK_a_	
	Measured	Reference		Measured	Reference
Barbituric acid	-3.31	/		3.81	4.01
Benzoic acid	-3.42	-3.36		4.05	4.18
Benzylamine	3.66	3.47		9.90	9.33
Phenol	-3.46	-3.44		10.03	9.99
m-Cresol	-3.34	-3.34		10.36	10.02

Reference data from Software PeakMaster 5.1; /: no data in the software database.

### 3.2 洛伐他汀*m*_0_和p*K*_a_的测定结果

采用DMSO作为电渗流标记物,利用一系列经验公式测定洛伐他汀的*m*_0_,利用得到的洛伐他汀*m*_0_值和线性回归直线进一步得到p*K*_a_值,分别为-1.70×10^-8^ m^2^/(V·s)和9.00。

本工作采用一系列不同离子强度的Na_2_HPO_4_-NaOH缓冲液(pH 12.00)测定洛伐他汀的*m*_0_。由前文可知,当溶液的pH值等于12.00时,比分析物的p*K*_a_值大3(p*K*_a_=9.00),此时*m*_act_几乎等于*m*_eff_,故式(6)适用于*m*_0_的计算。同时,如引言所述,当pH值大于7.7时,内酯型洛伐他汀会转化为酸型洛伐他汀,此时洛伐他汀的p*K*_a_应大于7.7。而测得的值为9.00,与假设相符,这说明结果具有较高的可靠性。

## 4 结论

本工作建立了毛细管电泳测定洛伐他汀的*m*_0_和p*K*_a_的方法。该方法基于毛细管区带电泳和离子淌度经验公式,且适用于酸性和碱性分析物p*K*_a_值的测定。同时,在pH值低于6的缓冲体系中,采用具有缩短迁移时间和检测无法到达阴极分析物的能力的反向毛细管电泳法测定p*K*_a_。通过与理论参考值对比可知,本文所建立的方法具有较高可靠度和准确性,这将在药物分析中发挥重要作用。本文所建立的方法简单快速,准确率高,能满足化合物*m*_0_和p*K*_a_的测定需要,同时也为测定化合物其他理化性质提供了参考依据。
